# Autophagy pathway induced by a plant virus facilitates viral spread and transmission by its insect vector

**DOI:** 10.1371/journal.ppat.1006727

**Published:** 2017-11-10

**Authors:** Yong Chen, Qian Chen, Manman Li, Qianzhuo Mao, Hongyan Chen, Wei Wu, Dongsheng Jia, Taiyun Wei

**Affiliations:** 1 Fujian Province Key Laboratory of Plant Virology, Institute of Plant Virology, Fujian Agriculture and Forestry University, Fuzhou, Fujian, PR China; 2 Fujian Key Laboratory for Monitoring and Integrated Management of Crop Pests, Institute of Plant Protection, Fujian Academy of Agricultural Sciences, Fuzhou, Fujian, PR China; Institute of Microbiology, CHINA

## Abstract

Many viral pathogens are persistently transmitted by insect vectors and cause agricultural or health problems. Generally, an insect vector can use autophagy as an intrinsic antiviral defense mechanism against viral infection. Whether viruses can evolve to exploit autophagy to promote their transmission by insect vectors is still unknown. Here, we show that the autophagic process is triggered by the persistent replication of a plant reovirus, rice gall dwarf virus (RGDV) in cultured leafhopper vector cells and in intact insects, as demonstrated by the appearance of obvious virus-containing double-membrane autophagosomes, conversion of ATG8-I to ATG8-II and increased level of autophagic flux. Such virus-containing autophagosomes seem able to mediate nonlytic viral release from cultured cells or facilitate viral spread in the leafhopper intestine. Applying the autophagy inhibitor 3-methyladenine or silencing the expression of *Atg5* significantly decrease viral spread *in vitro* and *in vivo*, whereas applying the autophagy inducer rapamycin or silencing the expression of *Torc1* facilitate such viral spread. Furthermore, we find that activation of autophagy facilitates efficient viral transmission, whereas inhibiting autophagy blocks viral transmission by its insect vector. Together, these results indicate a plant virus can induce the formation of autophagosomes for carrying virions, thus facilitating viral spread and transmission by its insect vector. We believe that such a role for virus-induced autophagy is common for vector-borne persistent viruses during their transmission by insect vectors.

## Author summary

Of the approximately 700 plant viruses, more than 75% are transmitted by insect vectors. However, the detailed mechanisms underlying the cellular responses induced by viral infection in insect vectors are poorly understood. We found that a plant reovirus could activate the autophagic process during persistent infection of leafhopper vectors. Furthermore, virus-induced autophagosomes can facilitate a nonlytic viral release and subsequent transmission by insect vectors. This work brings to a novel facet that a virus has evolved to activate and exploit autophagy to promote its transmission by insect vector, which may be a general mechanistic for vector-borne persistent viruses during their transmission by insect vectors.

## Introduction

Many viral pathogens that cause significant global health and agricultural problems are transmitted via insect vectors. To maximize transmission efficiency, viruses generally can modulate the biology and behavior of their vectors [1, 2]. Many arthropod-borne animal viruses (arboviruses) and plant viruses have evolved to be well adapted for persistent infection and maintenance in their insect vectors and may have some characteristics of insect pathogens [1, 2]. Such viruses circulate in the insect body and induce a variety of cellular responses that modulate the efficiency of viral transmission [2, 3]. However, the detailed mechanisms underlying the cellular responses induced by viral infection in insect vectors are poorly understood.

In mammals, viral infection can induce or activate autophagy, an important cellular response, which generally plays an important role against viruses [4, 5]. Autophagy is a highly conserved catabolic process that mediates the clearance of long-lived proteins and damaged organelles via a lysosomal degradative pathway [6, 7]. The mammalian target of rapamycin (mTOR) signaling pathways has been shown to control autophagy [9, 10]. These factors work in coordination to regulate autophagy, including the formation of autophagosomes and their fusion with lysosomes [4]. Under normal conditions, autophagy proceeds at a basal level, but it is significantly activated in response to a variety of stimuli, such as viral infection, nutrient starvation, and energy depletion [4, 11]. Although autophagy commonly serves as a defense mechanism against viral infection, some viruses appear to have evolved to exploit this mechanism to promote their survival and replication in different ways [12–23]. Thus, the role of autophagy in host-virus interactions is diverse for different viruses.

How autophagy induced by viral infection affects viral transmission by insect vectors is not well known. Previously, by using the model organism *Drosophila*, autophagy has been proved to play a direct antiviral role against the arbovirus vesicular stomatitis virus [24]. However, little is known about the role of autophagy in the interaction of insect vectors with plant viruses that they transmit. Recently, Wang et al. report that the begomovirus tomato yellow leaf curl virus (TYLCV) can activate autophagy in whitefly vectors to induce resistance to viral infection [25]. Thus, autophagy could serve as a defense mechanism against viral infection in insect vectors. Whether viruses can evolve to activate and exploit autophagy to promote their transmission by insect vectors is not well known.

In this study, we choose rice gall dwarf virus (RGDV), a plant reovirus, and its rice leafhopper vector *Recilia dorsalis* (Hemiptera: Cicadellidae) to determine how autophagy is activated to play a positive role in viral propagation and transmission by insect vectors. RGDV, which causes substantial yield loss in southern China and Southeast Asia, was first described in 1979 in Thailand [26]. RGDV propagates well and circulates in the body of its insect vector *R*. *dorsali* [27, 28]. Previously, we find that RGDV infection can directly remodel and utilize a variety of cellular structures and pathways for efficient propagation in its insect vector [29–31]. For example, RGDV particles are arrayed in an orderly manner close to the mitochondrial outer membrane during viral replication in vector cells, suggesting that mitochondria might support the energy demands of viral propagation in the insect vector [29]. Furthermore, RGDV particles are associated with microtubules either directly or via intermediate filaments to facilitate intracellular viral spread [30, 31]. By contrast, a conserved small interfering RNA (siRNA) antiviral response is triggered by RGDV infection to control viral propagation, avoiding excessive viral accumulation in insect vectors [32]. During viral infection in insect vector cells, abundant RGDV particles can be sequestered in vesicular compartments [2]. Similar vesicular compartments are involved in a nonlytic release of rice dwarf virus (RDV), also a plant reovirus, after fusion with the plasma membrane in virus-infected cultured leafhopper *Nephotettix cincticeps* cells [33–36]. In RGDV-infected leafhopper cells, we show that such compartments contain autophagy protein ATG8, indicating a potential role for a cellular autophagy pathway in nonlytic viral release. The subversion of the autophagic pathway by RGDV for *in vivo* viral spread also is investigated. Thus, we show that a plant virus can evolve to activate and exploit the autophagy to promote its transmission in insect vectors.

## Results

### RGDV infection activates the autophagy pathway in insect vector cells

**Fig 1:**
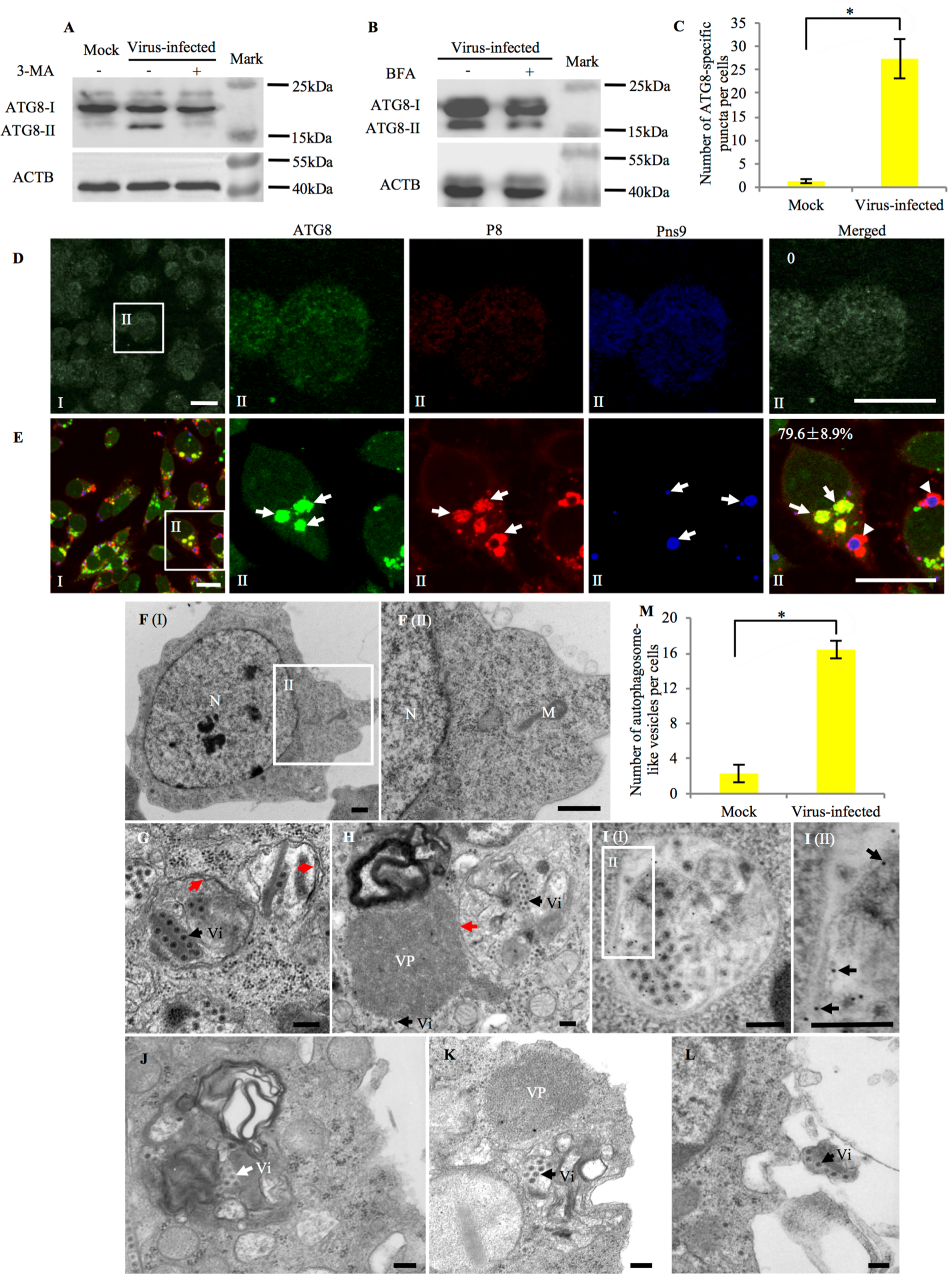
RGDV infection activated the autophagy pathway in VCMs of *R*. *dorsalis*. (A, B) At 48 hpi, ATG8 in mock- or virus-infected VCMs treated with (+) and without (-) 3-MA or BFA was detected by western blot assay using ATG8-specifc IgG. Insect ACTB was detected with ACTB-specific IgG as a control. (C-E) Autophagosomes in mock (D) and virus-infected (E) VCMs were detected by immunofluorescence microscopy. At 48 hpi, VCMs were immunolabeled for autophagosomes with ATG8-FITC (green), for viral particles with P8-rhodamine (red), and for viroplasms with Pns9-Alexa Fluor 647 (blue), and then processed for immunofluorescence microscopy. Viral antigens surrounding the spherical structures composed of Pns9, a component of viroplasms, are indicated by arrowheads. Arrows indicate the colocalization of ATG8-specific autophagosomes and viral particles. The average number of ATG8-specific puncta per *R*. *dorsalis* cell and a minimum of 100 cells were counted (C), and data points presented are the averages from six different fields (D, E). *P < 0.05. Bars, 15 μm. (F-L) Transmission electron micrographs for virus-induced autophagosomes in VCMs. Representative images were shown for mock VCMs (F), and for virus-containing autophagosomes in the cytoplasm (G) or at the periphery of viroplasm at 48 hpi (H). Panel II is an enlargement of the boxed area in panel I. Red arrows in panel G indicate double-membrane of autophagosomes. Red arrow in panel H indicates single-membrane of autophagosomes. (I) Virus-infected VCMs at 48 hpi were immunolabeled with ATG8-specific IgG as the primary antibody, followed by treatment with 10-nm gold particle-conjugated goat antibodies against rabbit IgG as secondary antibodies. Panel II is an enlargement of the boxed area in panel I. Arrows indicate gold particles. (J-L) At 48 hpi, virus-containing autophagosomes inside (J), at the periphery (K), or outside (L) of infected cells. (M) The quantification of the average number of autophagosome-like vesicles per *R*. *dorsalis* cell and a minimum of 30 cells were counted. *P < 0.05. M, mitochondrion. N, nucleus. Vi, virion. VP, viroplasm. Bars in panel F (I) and (II), 500 nm. Bars in panels G-L, 200 nm.

To determine whether the autophagy pathway can be induced upon RGDV infection, we first monitored the expression of 3 autophagy-related genes (*Ulk1/Atg1*, *Atg5* and *Atg8*) in continuous cultured vector cells in a monolayer (VCM) derived from *R*. *dorsalis* using an RT-qPCR assay. Our results showed that after inoculation with RGDV at a multiplicity of infection (MOI) of 0.4, the expression of 3 autophagy-related genes increased rapidly and significantly (*P* < 0.05) after 48 hours post inoculation (hpi) (S1 Fig). The autophagy-specific protein marker ATG8 is selectively enclosed within autophagosomes, and its breakdown allows measurement of the autophagic rate [8]. Thus, the conversion of ATG8-I to ATG8-II is generally considered to be a reliable indicator of autophagy [8, 25]. Here, we used ATG8-specific IgG to detect the autophagy pathway induced by RGDV infection in virus-infected *R*. *dorsalis* cells. At 48 hpi, the accumulation level of ATG8-II was increased notably in virus-infected VCMs (Fig 1A). We then used two inhibitors of autophagy, 3-methyladenine (3-MA) and Brefeldin A (BFA) [37, 38], to confirm RGDV infection activated the autophagy pathway. 3-MA inhibits autophagy due to the suppression of class III PtdIns 3-kinase [37], and BFA exerts its disruptive effect at the cis-Golgi, further demarcating a contribution of the Golgi with regard to autophagosome biogenesis [38]. At 48 hpi, we found that ATG8-II was almost lost after the treatment with 3-MA or BFA during viral infection of VCMs (Fig 1A and 1B), indicating that viral infection can induce the conversion of ATG8-I to ATG8-II. It seems that RGDV infection can induce the autophagy pathway in its insect vector cells.

We then used immunofluorescence microscopy to observe ATG8-specific autophagosomes in virus-infected VCMs. While nearly no autophagic signal was detected in uninfected VCMs, at 48 hpi, more than 20-fold ATG8-specific autophagosomes were found in RGDV-infected VCMs, and about 80% of them were colocalized with viral major outer capsid protein P8 (Fig 1C–1E), suggesting that viral particles may be enclosed by virus-induced autophagosomes. Rapamycin, an autophagy inducer that directly inhibits the action of TORC1 (target of rapamycin complex 1), was used as a positive autophagy indicator [4]. Confocal microscopy also showed that after rapamycin or ds*Torc1* treatment the level of autophagic signal was obviously detected in uninfected VCMs (S2 Fig). We further observed that ATG8-specific autophagosomes did not colocalize with the viroplasms of viral nonstructural protein Pns9 (Fig 1E), the site of viral replication and assembly of progeny virions [29, 30], suggesting that virus-induced autophagosomes were not the sites for viral replication. At 48 hpi, electron microscopy showed that virus-containing single- or double-membrane vesicular compartments appeared in the cytoplasm of virus-infected VCMs, in which cytosolic components or organelles were sequestered (Fig 1G and 1H). We observed that the number of double- or single- membrane vesicles increased more than 7-fold in the cytoplasm of virus-infected VCMs but rarely in virus-free VCMs (Fig 1M). Immunoelectron microscopy confirmed that ATG8-specifc IgG can specifically label such compartments, namely, autophagosomes in virus-infected VCMs, but did not recognize specific compartments in virus-free VCMs (Fig 1I). Thus, RGDV particles, assembled at the periphery of the viroplasm [29, 30], can be engulfed by virus-induced autophagosomes. Usually, these virus-containing autophagosomes were distributed within the cytoplasm (Fig 1J), at the periphery of cell membrane (Fig 1K) or outside the cells (Fig 1L). It seemed that virus-containing autophagosomes at the periphery of infected cells, can directly fuse with the plasma membrane to release viral particles (Fig 1K). Thus, virus-induced autophagy pathway may be involved in a nonlytic release of RGDV from insect vector cells.

### RGDV infection increases the levels of autophagic flux in insect vector cells

**Fig 2:**
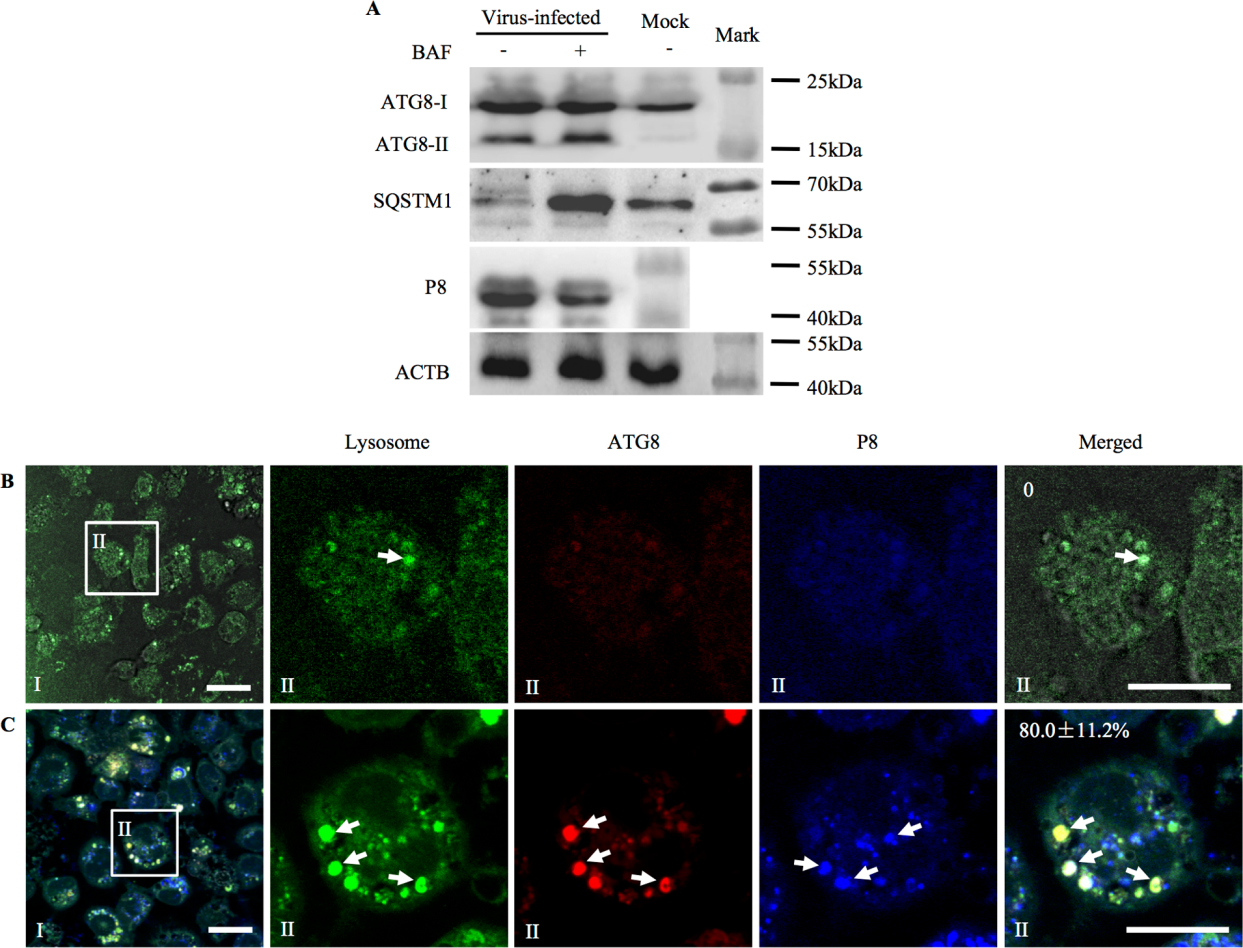
RGDV infection increased the level of autophagic flux in VCMs of *R*. *dorsalis*. (A) At 48 hpi, RGDV P8, ATG8 and SQSTM1 in VCMs treated with (+) and without (-) BAF were detected by western blot assay. Insect ACTB was detected with ACTB-specific IgG as a control. (B, C) Fusion of virus-induced autophagosomes with lysosomes in mock- (B) or virus-infected (C) VCMs, as revealed by immunofluorescence microscopy. At 48 hpi, VCMs were immunolabeled for lysosomes with LysoTracker Green DND-26 (green), for autophagosomes with ATG8-rhodamine (red), and for viral particles with P8-Alexa Fluor 647 (blue), and then processed for confocal microscopy. Data points presented are the averages from six different fields (B, C). Bars, 15 μm.

Autophagic flux is a continuous and complete process of autophagy by which lysosomes fuse with autophagosomes [4]. Autophagic adapter SQSTM1/p62 (sequestosome 1) is an indicator to assess autophagic flux because SQSTM1 can target specific cargo for autophagy and is specifically degraded by the autophagic-lysosomal pathway [4]. RT-qPCR assay showed that the expression of *Sqstm1* gene increased rapidly and significantly (*P* < 0.05) after inoculation with RGDV at a MOI of 0.4 (S1 Fig). However, at 48 hpi, western blot assay showed that the accumulation of SQSTM1 was significantly decreased during viral infection of VCMs (Fig 2A). We further revealed a clear correlation between the increase for the accumulation of ATG8-II and the decrease for the accumulation of SQSTM1 from 12 hpi to 96 hpi (S1 Fig). Thus, a clear autophagic flux occurred after viral infection of VCMs. Bafilomycin A1 (BAF) is a widely used inhibitor of the vacuolar type H^+^-ATPase (V-ATPase) that disturbs the fusion of autophagosomes with the lysosomes [39]. Next, the VCMs were treated with BAF, then inoculated with RGDV at a MOI of 0.4. At 48 hpi, we detected that the accumulation of SQSTM1 was significantly increased after BAF treatment during viral infection of VCMs (Fig 2A). Thus, viral infection may further cause the degradation of SQSTM1 by the autophagic-lysosomal pathway. However, BAF treatment would inhibit such virus-induced degradation process. Furthermore, western blot assay showed that the accumulation of viral major outer capsid protein P8 was substantially decreased after BAF treatment in virus-infected VCMs (Fig 2A). We thus determined that virus-induced autophagic flux was beneficial to viral infection, rather than serving as a defense mechanism for the degradation of viral particles.

Immunofluorescence microscopy further showed that LysoTracker-stained lysosomes were clearly found in RGDV-infected VCMs at 48 hpi, while uninfected VCMs exhibited almost no positive signs (Fig 2B and 2C). Furthermore, about 80% of LysoTracker-stained lysosomes were colocalized with viral major outer capsid protein P8 or ATG8-tagged autophagosomes (Fig 2C). Together, these results indicated that virus-containing autophagosomes were able to fuse with lysosomes, and insect vector cells underwent an autophagic process following RGDV infection.

### RGDV-induced autophagy pathway facilitates viral nonlytic release from insect vector cells

**Fig 3:**
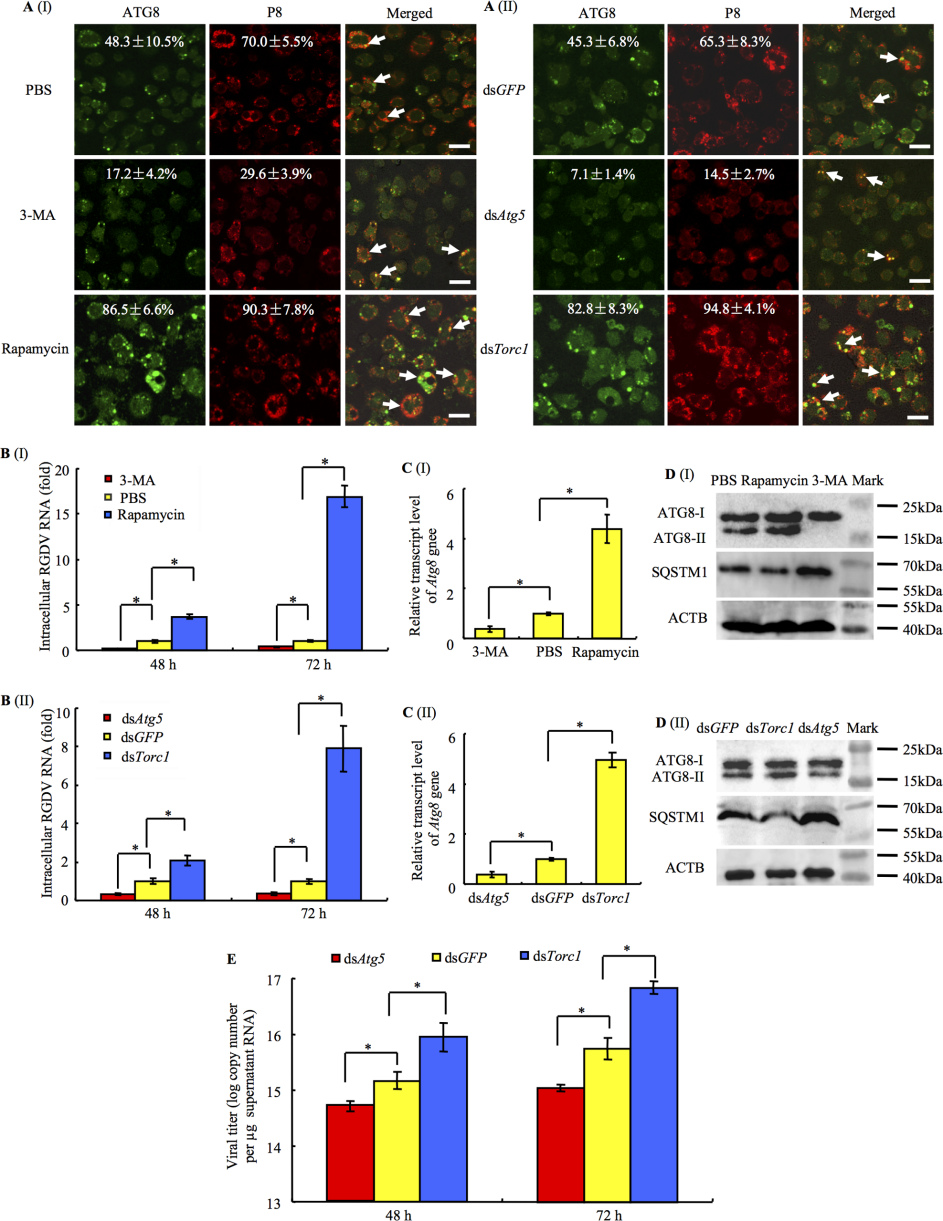
Autophagy pathway induced by RGDV infection facilitated viral nonlytic release from VCMs of *R*. *dorsalis*. (A) Percentage of insect vector cells infected with RGDV. The VCMs were transfected for 8 h with rapamycin or 3-MA (panels I) or ds*Atg5* or ds*Torc1* (panels II), then inoculated with RGDV at a MOI of 0.1 for 2 h. At 48 hpi, cells were immunolabeled with ATG8-FITC (green) and P8-rhodamine (red), then examined with confocal microscopy. Arrows indicate the colocalization of ATG8-specific autophagosomes and viral particles. Data points presented are the averages from six different fields (A). Bars, 20 μm. (B) At 48 and 72 hpi, effects of rapamycin or 3-MA (panels I) and ds*Atg5* or ds*Torc1* (panels II) on transcript levels of RGDV P8 gene in VCMs as revealed by RT-qPCR assay. Means (±standard deviation [SD]) from three biological replicates are shown. *P < 0.05. (C) At 48 hpi, effects of rapamycin or 3-MA (panels I) and ds*Atg5* or ds*Torc1* (panels II) on transcript levels of *Atg8* gene in VCMs as revealed by RT-qPCR assay. Means (±SD) from three biological replicates are shown. *P < 0.05. (D) The accumulation levels of ATG8 and SQSTM1 were detected by western blot assay using ATG8- and SQSTM1-specifc IgG, respectively. Insect ACTB was detected with ACTB-specific IgG as a control. (E) Autophagy induced by viral infection increased the extracellular viral RNA levels. VCMs were transfected for 8 h with dsRNAs, then inoculated with RGDV at a MOI of 10 for 2 h. At 48 and 72 hpi, culture supernatant was collected to measure the viral titers detected by RT-qPCR assay. Means (±SD) from three biological replicates are shown. *P < 0.05.

To confirm the existence of such a viral release pathway by virus-induced autophagosomes, we studied the effects of inhibitors or inducers of autophagy applied during viral infection in VCMs. We inhibited or induced autophagy by the drugs 3-MA or rapamycin, respectively. We also silenced the expression of *Atg5* or *Torc1* genes by RNA interference (RNAi) to inhibit or induce autophagy, respectively [4, 9, 40]. VCMs were treated with drugs (3-MA or rapamycin) or dsRNAs targeting to *Atg5*, *Torc1* or *GFP* genes (ds*Atg5*, ds*Torc1* or ds*GFP*). At 8 h after treatment, the treated VCMs were inoculated with RGDV at a MOI of 0.1. At this low MOI, the early viral infection rate was low (about 15–30%), and the spread of viruses among VCMs could be easily monitored. By 48 hpi, immunofluorescence microscopy indicated that the treatment with *dsTorc1* or rapamycin increased the percentage of infected cells from an average of 65% or 70% to 95% or 90% when compared with ds*GFP*- or PBS-treated cells, respectively (Fig 3A). In contrast, treatment with ds*Atg5* or 3-MA decreased the percentage of infected cells from an average of 65% or 70% to 15% or 30% when compared with the ds*GFP*-or PBS-treated cells, respectively (Fig 3A). As expected, ATG8-specific autophagosomes were clearly observed in virus-infected regions, but not in virus-free regions (Fig 3A). RT-qPCR assay showed that the treatment with ds*Torc1* or rapamycin increased viral titers by about 2–17-fold (Fig 3B). By contrast, the treatment with ds*Atg5* or 3-MA significantly reduced viral titers (Fig 3B). Thus, viral infection was positively correlated to autophagosome formation. We confirmed that the transcript levels of *Atg8* gene were significantly reduced by treatment with 3-MA or ds*Atg5*, but was increased after treatment with rapamycin or ds*Torc1* (Fig 3C). Accordingly, western blot revealed that the treatment with 3-MA or ds*Atg5* reduced the accumulation of ATG8-II, but the treatment with rapamycin or ds*Torc1* increased the accumulation of ATG8-II in virus-infected VCMs (Fig 3D). In addition, there was a clear correlation between the increase for ATG8-II accumulation and the decrease for SQSTM1 accumulation (Fig 3D). These observations indicated that the inducing of autophagy would facilitate viral infection, while the blocking of autophagy will inhibit viral infection. Thus, autophagy is beneficial to viral infection in insect vector cells.

We further determined whether the autophagy pathway triggered by viral infection can facilitate viral release from insect vectors. VCMs were treated with ds*Torc1* or ds*Atg5*. At 8 h after treatment, the treated VCMs were inoculated with RGDV at a MOI of 10 to guarantee that the early viral infection rate was high (100%). The extracellular medium was collected, and the viral titer was quantified by RT-qPCR assay. As expected, the treatment with ds*Torc1* significantly increased viral titers in the medium, whereas ds*Atg5* decreased titers (Fig 3E). All these results suggested that the autophagy pathway induced by viral infection was necessary for the release of RGDV into the cell culture medium.

### RGDV infection activates the autophagy pathway in the intestines of *R*. *dorsalis*

**Fig 4:**
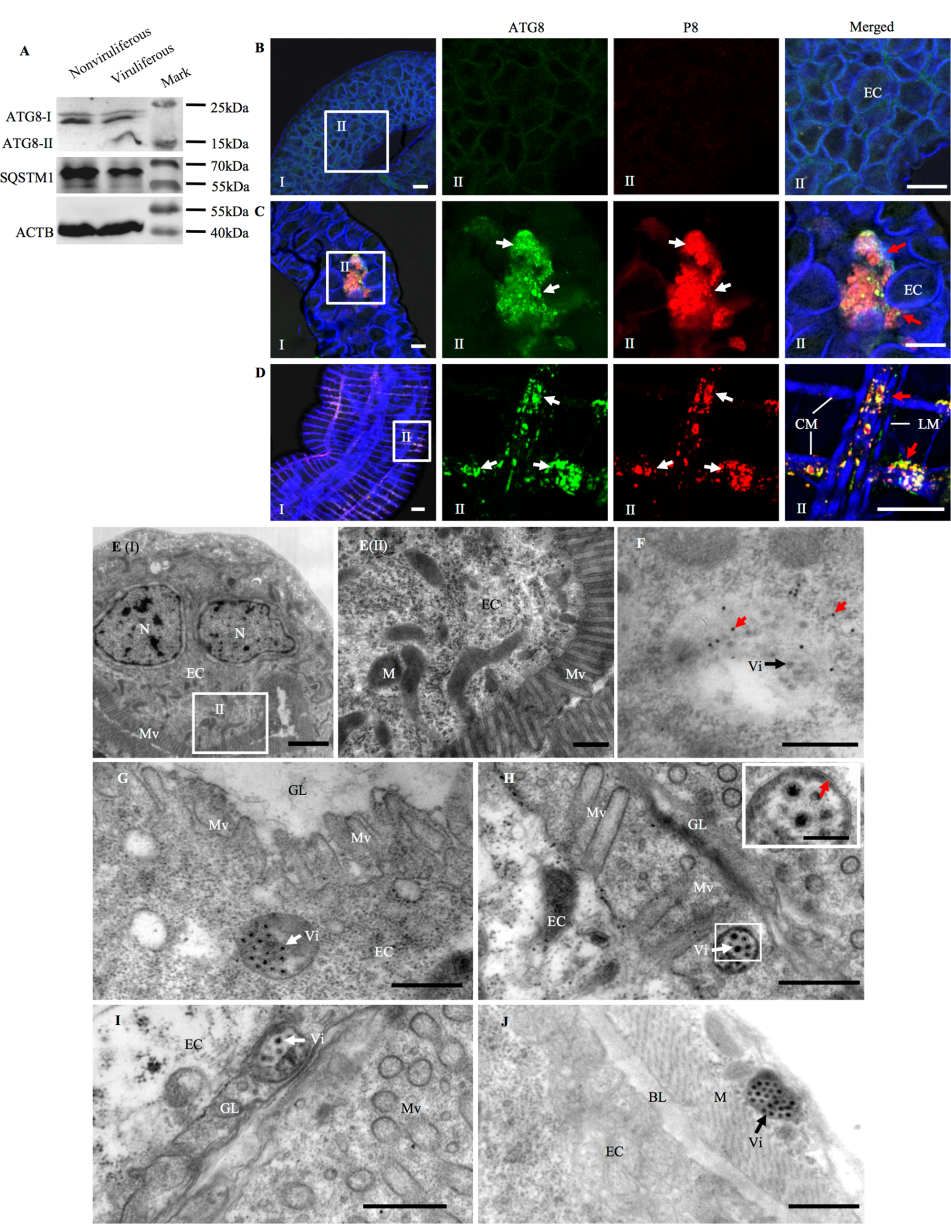
RGDV infection triggered autophagy pathway in the intestine of *R*. *dorsalis*. (A) At 4 days padp, ATG8 and SQSTM1 in nonviruliferous or viruliferous insects were detected by western blot assay using ATG8- and SQSTM1-specifc IgG, respectively. Insect ACTB was detected with ACTB-specific IgG as a control. ATG8-I was detected in both nonvirulifeorus and viruliferous insects, and ATG8-II was only detected in viruliferous insects. (B-D) Autophagosomes in nonviruliferous (B) or viruliferous (C, D) insects were detected by confocal microscopy. At 4 days padp, insect intestines were immunolabeled for autophagosomes with ATG8-FITC (green), for viral particles with P8-rhodamine (red), and for ACTB with ACTB dye phalloidin-Alexa Fluor 647 (blue), then examined with confocal microscopy. Panels II are enlargements of boxed areas in panels I. Single optical sections of epithelial side (C) and muscle side (D) of the intestines from viruliferous *R*. *dorsalis* are shown from a confocal *Z*-stack at 100 μm depth. Red arrows indicate colocalization of ATG8-specific autophagosomes and P8-specific viral particles. Bars, 30 μm. (E-J) RGDV infection induced autophagosome formation as measured by electron microscopy. (E) Representative images were shown for nonviruliferous intestinal epithelium. Panel II was an enlargement of the boxed area in panel I. (F) Viruliferous intestinal epithelium was immunolabeled with ATG8-specific IgG as the primary antibody, followed by treatment with 10-nm gold particle-conjugated goat antibodies against rabbit IgG as secondary antibodies. Red arrows indicate gold particles. (G-J) Transmission electron micrographs of virus-containing autophagosomes within the epithelial cytoplasm (G), along the microvilli (H), in the gut lumen (I), or in the visceral muscles tissues (J) of insect intestines. Inset in panel H was the enlargement of the boxed area. Red arrow indicated the double-membrane of virus-containing autophagosome. Vi, virion. CM, circular muscle. LM, longitudinal muscle. BL, basal lamina. Mv, microvilli. M, mitochondrion. N, nucleus. EC, epithelial cell. GL, gut lumen. Bar in panel E (I), 2 μm. Bars in panels E (II)-J, 500 nm. Bar in inset of panel H, 200 nm.

To determine whether autophagy is triggered upon RGDV infection in the body of insect vectors *in vivo*, ATG8-specific IgG was used to detect virus-induced autophagosomes in viruliferous *R*. *dorsalis*. RT-qPCR assay indicated that the expression of 3 autophagy-related genes (*Ulk1*, *Atg5* and *Atg8*) and *Sqstm1* gene increased significantly (*P* < 0.05) in viruliferous leafhoppers (S1 Fig). Western blot assay showed that ATG8-II can be specifically detected in the viruliferous, but not in the nonviruliferous *R*. *dorsalis* (Fig 4A). Furthermore, the accumulation of SQSTM1 was decreased notably in viruliferous *R*. *dorsalis* (Fig 4A). There was a clear correlation between the increase for ATG8-II accumulation and the decrease for SQSTM1 accumulation as well during viral infection of insect vectors (S1 Fig). Together, these results indicated that RGDV induced the autophagy activity in *R*. *dorsalis*.

To further verify that the autophagy pathway was activated by RGDV infection, the formation of autophagosomes in the intestine of *R*. *dorsalis* was examined using immunofluorescence microscopy. No specific labeling of ATG8 was detected in the intestines of nonviruliferous *R*. *dorsalis* (Fig 4B). At 4 days post-first access to diseased plants (padp), we observed that ATG8-specific autophagosomes colocalized with P8 of RGDV in the epithelium and visceral muscles of the intestine in viruliferous *R*. *dorsalis* (Fig 4C and 4D). Immunoelectron microscopy confirmed that ATG8-specifc IgG can specifically label virus-containing autophagosomes in the intestinal epithelium (Fig 4F). Electron microscopy further revealed that virus-containing single- or double-membrane autophagosomes distributed in the epithelium cytoplasm (Fig 4G), the microvilli (Fig 4H) and the gut lumen (Fig 4I). Thus, RGDV may exploit these autophagosomes to move from the intestinal epithelium to the gut lumen through the microvilli. Furthermore, RGDV may also use such autophagosomes to move along actin filaments to spread through the visceral muscles (Fig 4J). Together, our results suggested that RGDV activated the autophagy pathway in *R*. *dorsalis* to mediate viral release from the intestinal epithelium.

### RGDV-induced autophagy pathway facilitates viral infection and transmission by *R*. *dorsalis*

**Fig 5:**
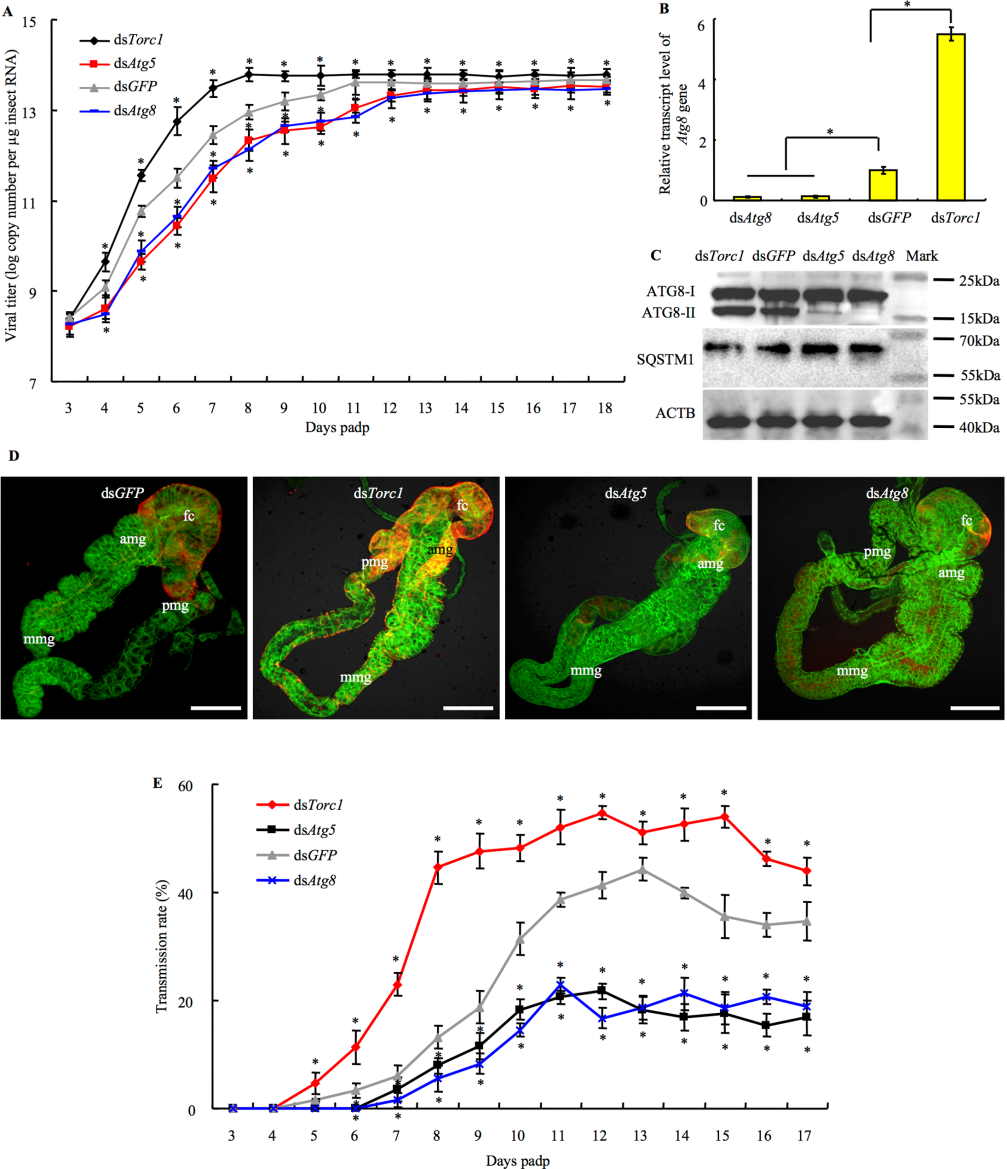
Autophagy pathway induced by RGDV infection facilitated viral spread in the intestine of *R*. *dorsalis*. (A) At different days padp, 30 live dsRNAs-treated leafhoppers positive for transcript of RGDV P8 gene were used for assay of viral genome copies, which were calculated as the log of the copy number of P8 gene/μg insect RNA. Means (±SD) from three biological replicates are shown. The statistical significance was related to the ds*GFP* control. *P < 0.05. (B) At 48 hpi, effects of ds*Atg5*, ds*Atg8* or ds*Torc1* on transcript levels of *Atg8* gene in dsRNAs-treated leafhoppers as revealed by RT-qPCR assay. ACTB was used as the internal control. Means (±SD) from three biological replicates are shown. *P < 0.05. (C) The accumulation levels of ATG8 and SQSTM1 in dsRNAs-treated leafhoppers were detected by western blot assay using ATG8- and SQSTM1-specifc IgG, respectively. Insect ACTB was detected with ACTB-specific IgG as a control. (D) RGDV infection in the intestine of leafhoppers receiving dsRNAs at 4 days padp, as detected by immunolabelling with P8-rhodamine (red) and the ACTB dye phalloidin-FITC (green). Bars, 200 μm. (E) Effects of autophagy pathway on the transmission of RGDV by *R*. *dorsalis*. Leafhoppers treated with dsRNAs were used to infect susceptible rice plants, and viral transmission rate was determined by RT-PCR assay. Means (±SD) from three independent replicates are shown. The statistical significance is related to the ds*GFP* control. *P < 0.05. fc, filter chamber. mg, midgut. amg, anterior midgut. mmg, middle midgut. pmg, posterior midgut.

To confirm whether the autophagy pathway promoted viral infection in the insect body, from 3 to 18 days padp, we daily sampled 30 live viruliferous leafhoppers that microinjected with ds*Atg5*, ds*Atg8*, ds*Torc1* or ds*GFP*, then calculated viral genome copies for the major outer capsid protein P8 gene of RGDV. RT-qPCR assay showed that the mean viral genome copies in dsRNAs-treated viruliferous leafhoppers increased rapidly before 8–10 days padp, and then remained nearly stable (Fig 5A). Previously, we have shown that RGDV infection in insect vectors triggered a strong siRNA antiviral response, which can efficiently control viral accumulation below the pathogenic threshold to maintain the persistent infection [32]. Thus, our results were consistent with the persistent infection pattern of RGDV in insect vectors. It was clear that the mean viral genome copies in ds*Torc1*-treated viruliferous leafhoppers were significantly (*P* < 0.05) higher than those in ds*GFP*-treated controls (Fig 5A). By contrast, the mean viral genome copies in ds*Atg5*- or ds*Atg8*-treated viruliferous leafhoppers were significantly (*P* < 0.05) lower than those in ds*GFP*-treated controls (Fig 5A). We confirmed that the transcript levels of *Atg8* gene were significantly reduced by treatment with ds*Atg8* or ds*Atg5*, but was increased after treatment with ds*Torc1* in viruliferous leafhoppers (Fig 5B). Furthermore, western blot assay confirmed that ds*Atg5* or ds*Atg8* treatment reduced ATG8-II accumulation, but increased SQSTM1 accumulation in viruliferous leafhoppers (Fig 5C). However, ds*Torc1* treatment increased ATG8-II accumulation, but reduced SQSTM1 accumulation in viruliferous leafhoppers (Fig 5C). Taken together, our results suggested that the activation of autophagy by ds*Torc1* treatment facilitated viral accumulation, whereas inhibiting of autophagy by ds*Atg5* or ds*Atg8* treatment blocked viral accumulation in insect vectors.

Using immunofluorescence microscopy at 4 days padp, we also examined individual intestines of *R*. *dorsalis* that had been treated with dsRNAs. Our results showed that RGDV accumulated in a particular corner of the filter chamber after the ds*Atg5* or ds*Atg8* treatment (Fig 5D). However, in the ds*Torc1* treatment, RGDV spread from the filter chamber into the adjacent midgut regions (Fig 5D). In ds*GFP*-treated leafhoppers, RGDV had spread from the filter chamber into the visceral muscles (Fig 5D), confirming that the autophagy promoted viral infection.

We next determined whether virus-induced autophagy pathway facilitated the transmission of RGDV via insect vectors to rice plants. Individual leafhoppers microinjected with dsRNAs were fed on individual rice seedlings in individual tubes to test the transmission rates, which were calculated based on the number of virus-infected rice plants/total number of rice plants tested. We found that the transmission rates for dsRNA-treated viruliferous leafhopper increased steadily from 5 to 8 or 10 days padp, and then remained stable from 8 or 10 to 17 days padp (Fig 5E). Compared with the ds*GFP* treatment, the ds*Torc1* treatment considerably increased the transmission rates from 5 to 17 days padp, whereas ds*Atg5* or ds*Atg8* treatment significantly compromised leafhopper ability to transmit the virus to rice seedlings from 6 to 17 days padp (Fig 5E). Together, these data demonstrated that the autophagy pathway facilitated viral transmission efficiency by *R*. *dorsalis*.

To determine whether RGDV also induce autophagy pathway in rice hosts. We inhibited autophagy in rice plant by the drug 3-MA. We found that the transcript levels of *Atg8* gene were significantly reduced by 3-MA treatment. However, viral genome copies in 3-MA -treated rice plants were no statistical differences (*P* > 0.05) compared with the PBS-treated controls (S3 Fig). Furthermore, no virus-containing autophagosomes were observed in virus-infected rice plants by electron microscopy (S3 Fig). Thus, it seemed that RGDV did not induce autophagy pathway in rice hosts.

### RDV infection also activates the autophagy pathway in its insect vector

**Fig 6:**
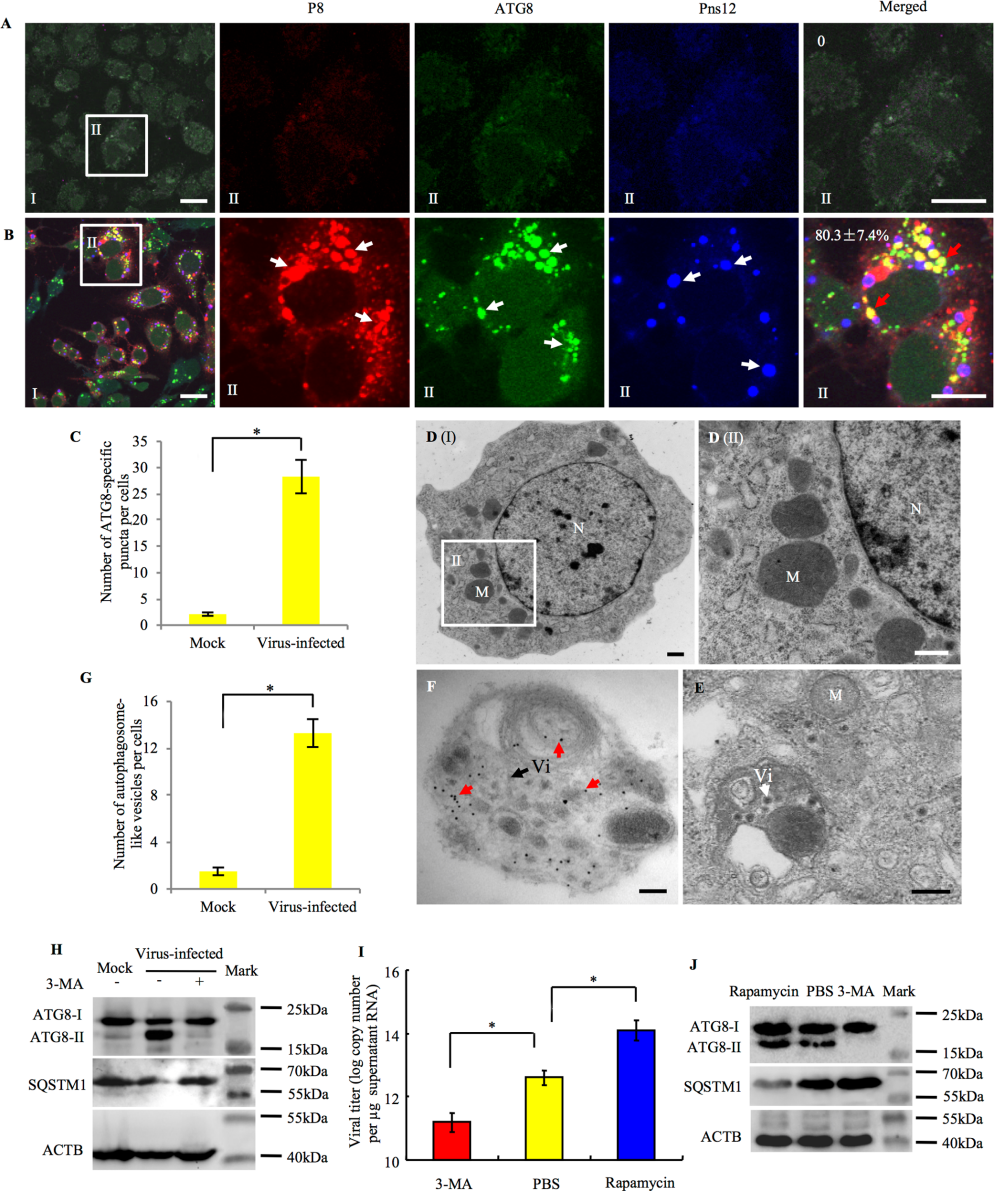
RDV infection activated autophagy pathway in VCMs of *N*. *cincticeps*. (A, B) At 48 hpi, mock- or virus-infected VCMs were immunolabeled for autophagosomes with ATG8-FITC (green), for viral particles with P8-rhodamine (red), and for viroplasms with Pns12-Alexa Fluor 647 (blue), and then processed for confocal microscopy. Red arrows indicate colocalization of ATG8-specific autophagosomes and P8 antigens of RDV. Data points presented are the averages from six different fields. Bars, 10 μm. (C) The average number of ATG8-specific puncta per *N*. *cincticeps* cell and a minimum of 100 cells were counted. *P < 0.05. (D-F) Transmission electron micrographs for virus-induced autophagosomes in VCMs. (D) Representative images were shown for mock VCMs. Panel II was an enlargement of the boxed area in panel I. Bars, 500 nm. (E) Electron microscopy showed the presence of virus-containing autophagosomes in the cytoplasm. (F) Immunoelectron micrographs of virus-containing autophagosomes positive for ATG8. VCMs were immunolabeled with ATG8-specific IgG as the primary antibody, then treated with 15-nm gold particle-conjugated goat antibodies against rabbit IgG as secondary antibodies. Red arrows indicate gold particles. Bars in panels E-F, 200 nm. (G) The quantification of the average number of autophagosome-like vesicles per *N*. *cincticeps* cell and a minimum of 30 cells were counted. *P < 0.05. (H) At 48 hpi, ATG8 and SQSTM1 in VCMs treated with (+) and without (-) 3-MA were detected by western blot assay, respectively. Insect ACTB was detected with ACTB-specific IgG as a control. (I) Autophagy induced by viral infection increased the extracellular viral RNA levels. VCMs were transfected for 8 h with 3-MA or rapamycin, then inoculated with RDV at a MOI of 10 for 2 h. At 48 hpi, culture supernatant was collected to measure the viral titers detected by RT-qPCR assay. Means (±SD) from three biological replicates are shown. *P < 0.05. (J) At 48 hpi, the accumulation levels of ATG8 or SQSTM1 in 3-MA- or rapamycin-treated VCMs were analyzed by western blot assay. Insect ACTB was detected with ACTB-specific IgG as a control. M, mitochadria. N, nucleus. Vi, virion.

RDV, a plant reovirus closely related to RGDV, is also transmitted by rice leafhopper *N*. *cincticeps*. Immunofluorescence microscopy showed that virus-containing vesicular compartments were positive for the autophagy marker ATG8 in RDV-infected VCMs (Fig 6A and 6B). In contrast to the virus-free cells, it appeared that more than 15-fold ATG8-specific puncta in RDV-infected VCMs, and about 80% of ATG8-specific autophagosomes can colocalize with viral major outer capsid protein P8, but they never were overlapped with viroplasms of viral nonstructural protein Pns12 (Fig 6A–6C). Previously, we had shown that progeny virions that assembled at the periphery of viroplasm can be sequestered into vesicular compartments, which would mediate nonlytic viral release from insect vector cells [34–36]. We observed that the number of double- or single- membrane vesicles increased more than 9-fold in the cytoplasm of virus-infected VCMs but rarely in virus-free VCMs (Fig 6D, 6E and 6G). Furthermore, immunoelectron microscopy further indicated that ATG8-specific IgG can specifically recognize such virus-containing vesicular compartments, namely, autophagosomes (Fig 6F). Similar to RGDV, western blot assay also showed that RDV infection activated the appearance of ATG8-II, but reduced SQSTM1 accumulation (Fig 6H). An autophagy inhibitor, 3-MA, strongly inhibited the conversion of ATG8-I to ATG8-II and the degradation of SQSTM1 during viral infection (Fig 6H). Thus, we determined that RDV infection also triggered the autophagy pathway in its insect vector cells.

We further determine whether the autophagy pathway triggered by RDV infection also facilitate viral spread among insect vector cells. After treated with 3-MA or rapamycin, VCMs were inoculated with RDV at a MOI of 10. At 48 hpi, RT-qPCR assay showed that the treatment with rapamycin significantly increased viral titers, whereas the treatment with 3-MA decreased viral titers in the medium (Fig 6I). Western blot assay further confirmed that rapamycin promoted the conversion of ATG8-I to ATG8-II and the degradation of SQSTM1 (Fig 6J). Thus, the inhibiting of virus-induced autophagy blocked vial release, whereas the activating of virus-induced autophagy facilitated viral release form insect vector cells. Our results revealed a common mechanism for plant reoviruses to induce autophagy for viral efficient spread in insect vector cells.

## Discussion

Viruses can induce or activate cellular responses such as apoptosis or autophagy to facilitate viral infection cycle in hosts or vectors [41, 42]. Here, we demonstrated that infection by the plant reovirus RGDV significantly triggered an increase in virus-containing single- or double-membrane autophagosomes, the colocalization of ATG8-II with viral particles, and the conversion of ATG8-I to ATG8-II in virus-infected *R*. *dorsalis* cells (Fig 1), indicating that autophagy pathway was activated by RGDV infection in insect vector cells. We further showed that RGDV infection promoted the degradation of autophagic adapter SQSTM1 and caused the fusion of virus-containing autophagosomes with lysosomes (Figs 2 and S1). The treatment of lysosome inhibitor BAF suppressed such degradation of SQSTM1 during viral infection (Fig 2). Thus, the autophagic flux was triggered by RGDV infection. By inhibiting or activating autophagy with chemical reagents 3-MA and rapamycin or by RNAi induced by dsRNAs targeting *Atg5* or *Torc1* genes, we demonstrated a proviral role for virus-induced autophagy pathway in RGDV release from insect vector cells (Fig 3). In our electron micrographs, progeny RGDV virions assembled at the periphery of viroplasms [29, 30], were engulfed by virus-induced autophagosomes, which then evidently mediated nonlytic viral release by fusion with the plasma membrane in insect vector cells (Fig 1). Thus, RGDV infection activated the autophagy pathway, which facilitated viral spread rather than controlling viral infection in insect vector cells. We further showed that the plant reovirus RDV also induced the autophagy pathway and subsequently mediated nonlytic viral release from its *N*. *cincticeps* vector cells (Fig 6). Generally, plant reoviruses in insect vector cells are sequestered in spherical vesicular compartments [2]. We thus deduced that the exploitation of virus-induced autophagy pathway for viral spread among insect vector cells may be a conserved mechanism for plant reoviruses.

Previously, we have shown that plant reoviruses enter insect vector cells through a receptor-mediated, clathrin-dependent endocytosis, and then are sequestered in the early endosomes [34]. The low pH in the early endosomes is necessary for the proteolytic processing of reovirus outer capsid proteins, which is the essential step for the early stage of viral infection [43–45]. Our current results showed that, after viral replication and assembly of progeny virions, plant reoviruses can be sequestered in the autophagosomes or lysosomes. This later infection event is quite different from the early entry stage of plant reoviruses. Thus, the proteolytic processing of reovirus outer capsid proteins may not occur in the virus-induced autophagosomes or lysosomes at the later infection stage of plant reoviruses in insect vectors cells.

Generally, autophagy is an important antiviral cellular response for degradation of viral proteins or interference with viral replication [46]. On the other hand, as a result of continuous coevolution, many arboviruses have developed sophisticated mechanisms to subvert autophagy pathway and thus promote different stages of viral life cycle in their mammalian hosts [47–52]. However, until the present study, the role of autophagy in natural interactions of arboviruses with their insect vectors has been less studied. In *Drosophila*, autophagy-induced by arboviruses was considered as an antiviral immunity response [24]. So far, only one study has considered a potential antiviral role for autophagy in plant virus-insect vector system. Wang et al. reported that a single-stranded DNA plant virus TYLCV can activate the whitefly autophagy pathway, which leads to subsequent degradation of the virus *in vivo* [25]. However, TYLCV-induced autophagosomes do not contain viral particles or capsids [25]. Here, we showed that the persistent infection of RGDV can trigger the accumulation of abundant virus-containing autophagosomes in the intestine epithelium of leafhopper vector (Fig 4). However, it is clear that RGDV can escape lysosomal degradation and exploit such autophagosomes to release from intestinal epithelium into the lumen by passing through actin-based microvilli (Fig 4). Furthermore, such autophagosomes may move along actin-based visceral muscles surrounding the intestinal epithelium (Fig 4). Thus, we demonstrated that virus-induced autophagy pathway played a critical role in viral spread in vector insects, enabling to accomplish a latent period for the virus, and subsequent ability to transmit the virus to plant hosts. Similarly, in cultured mammalian cells, a potential role for autophagy pathway in nonlytic release of human poliovirus or hepatitis A virus *in vitro* has been demonstrated [52–54]. However, little is known about the mechanisms of these human viruses spread via the internal mammalian host tissues such as the intestine, muscle tissue, and peripheral neurons during a natural infection. Our novel model for virus-induced autophagy pathway exploited by a virus to spread in insect intestine may be a common mechanism of spread for other viruses *in vivo*. We believe that such a role for virus-induced autophagy pathway is common for vector-borne persistent viruses during their transmission by insect vectors.

Plant reoviruses, once ingested by the insects, establish their primary infection in a limited number of intestinal epithelial cells, then the invading viruses can initiate the formation of nascent viroplasms for viral multiplication [1–2]. Later, the progeny virions directly crossed the basal lamina into the visceral muscles, and spread into the salivary glands to be horizontally transmitted to healthy plants or into the female ovary to be vertically transmitted to offspring [1–2]. Here, we demonstrated that autophagosomes induced by plant reoviruses assist viral particles pass through the membrane barriers, facilitating viral transmission. However, one key question remains unanswered to understand this model. Why do plant reoviruses such as RGDV and RDV induce the autophagy pathway for viral spread in insect vectors? Our recent study shows that a conserved siRNA antiviral immunity response is triggered by persistent replication of plant reoviruses in their insect vector [32, 55]. We thus deduced that the autophagosomes may be exploited by plant reoviruses to escape the direct defense from virus-induced siRNA antiviral immunity responses. Furthermore, the membrane structure of virus-induced autophagosome is a useful vehicle to carry virions to overcome any membrane or tissue barriers in insect vectors. Previously, we show that plant reoviruses can exploit the tubules constituted by viral nonstructural proteins to pass through the actin-based microvilli of the intestine epithelium either into the lumen or across the basal lamina into the circular visceral muscle of insect vectors [31, 33, 56, 57]. Based on these considerations, we deduce that, in an apparent trade-off between plant reoviruses and insects, the autophagy pathway and other modes of cellular remodeling are induced to facilitate viral accumulation, whereas the insect’s innate immune responses, such as the siRNA antiviral pathway, are induced to maintain viral accumulation below the pathogenic threshold [32, 55]. Thus, the two mechanisms for the spread of plant reoviruses in insect vectors not only facilitate rapid viral dissemination but may also promote evasion of immune defenses, guaranteeing that the virus can be transmitted with high efficiency while maintaining a persistent infection that is not lethal.

## Methods

### Insects, cells, virus and antibodies

Leafhoppers (*R*. *dorsalis* and *N*. *cincticeps*) were collected from Guangdong Province in southern China. VCMs derived from *R*. *dorsalis* and *N*. *cincticeps* were maintained on the growth medium as described previously [58]. The RGDV and RDV isolates were maintained on rice plants via transmission by *R*. *dorsalis* and *N*. *cincticeps*, respectively, as reported previously [59]. The major outer capsid protein P8 and the nonstructural protein Pns9 of RGDV, as well as the major outer capsid protein P8 and the nonstructural protein Pns12 of RDV were prepared as described previously [33, 36]. IgGs, isolated from the polyclonal antibodies were conjugated to fluorescein isothiocyanate (FITC), rhodamine or Alexa Fluor 647 carboxylic acid (Invitrogen) according to the manufacturer’s instructions.

### Detection of virus-induced autophagy pathway by immunofluorescence microscopy

VCMs derived from *R*. *dorsalis* growing on a coverslip were inoculated with RGDV at a MOI of 0.4 or 1.0 for 2 h as described previously [32]. At 48 hpi, VCMs were immunolabeled for autophagosomes with ATG8-specific IgG (prepared by our laboratory) conjugated to FITC (ATG8-FITC), for viral particles with P8-specific IgG (prepared by our laboratory) conjugated to rhodamine (P8-rhodamine), and for viroplasms with Pns9-specific IgG (prepared by our laboratory) conjugated to Alexa Fluor 647 carboxylic acid (Pns9-Alexa Fluor 647), and then processed for immunofluorescence microscopy as already described [57]. For lysosome staining, the mock- or virus-infected VCMs were treated with 1 μM LysoTracker (Green DND-26, Invitrogen) at 37°C for 30 min [60]. At 48 hpi, VCMs were fixed, immunolabeled with ATG8-FITC and P8-specific IgG conjugated to Alexa Fluor 647 carboxylic acid (P8-Alexa Fluor 647), and then processed for immunofluorescence microscopy, as already described [32, 60]. To detect whether RDV infection induced autophagy pathway in insect vector cells, VCMs derived from *N*. *cincticeps* growing on a coverslip were inoculated with RDV at a MOI of 0.4 for 2 h. At 48 hpi, VCMs were immunolabeled with ATG8-FITC, P8-rhodamine, Pns12-specific IgG conjugated to Alexa Fluor 647 carboxylic acid (Pns12-Alexa Fluor 647), and then processed for immunofluorescence microscopy (Leica TCS SP5 II) as already described [57].

Immunofluorescence labeling of the intestine of *R*. *dorsalis* after acquisition of RGDV from diseased rice plants was described as previously [57]. Second *R*. *dorsalis* instars were fed on diseased rice plants for 1 day and then transferred to healthy rice seedlings. At 4 days padp, the intestines were dissected, fixed, immunolabeled with ATG8-FITC, P8-rhodamine and actin dye phalloidin-Alexa Fluor 647 carboxylic acid (Invitrogen), and then processed for immunofluorescence microscopy, as described previously [28, 32].

Cells containing more than two ATG8-speific puncta were defined as autophagy-positive cells. The number or the percentage of the cells showing the ATG8-positive signs were counted under a fluorescence microscope [50].

### Detection of virus-induced autophagy pathway by electron microscopy

Virus-infected rice plants, VCMs on coverslips or insect intestines were fixed, dehydrated and embedded and thin sections cut as described previously [57, 58]. Sections were then incubated with ATG8-specific IgG and immunogold labelled with goat antibodies against rabbit IgG which had been conjugated with 10- or 15-nm-diameter gold particles (Sigma) [57, 58]. The average number of autophagic vesicle (AV) per cell was evaluated. A minimum of 30 cells were observed. Cell counting was done by three independent experiments and data are presented as mean ± standard deviation [SD].

### Detection of virus-induced autophagy pathway by western blot assay

Total proteins from VCMs or intact insects were extracted using the sample buffer and separated by 10 or 12% SDS-PAGE, then transferred to polyvinylidine difluoride membranes (Bio-Rad). The membranes were blocked with 5% nonfat milk in PBS with 0.1% Tween 20 and then incubated with RGDV P8-specific IgG, ATG8-specific IgG, ACTB-specific IgG (Purchased from Sigma), or SQSTM1-specific IgG (Purchased from Cell Signal Technology). After incubation with secondary antibody (MultiSciences Biotech), proteins were visualized with the Luminata Classico Western HRP Substrate (Millipore) and imaged with the Molecular Imager ChemiDoc XRS+ System (Bio-Rad).

To confirm RGDV or RDV infection activated the autophagy pathway, autophagy inhibitors (100 nM 3-MA, Sigma; 1 μM BFA, Selleckchem; 20 nM BAF, Enzo) were used to treat the VCMs to inhibit autophagosome formation. VCMs were transfected with (+) and without (-) 3-MA, BFA or BAF for 8 h, and then inoculated with RGDV or RDV at a MOI of 1.0 for 2 h. At 48 hpi, the cells were collected for western blot assay.

### Detection of virus-induced autophagy pathway by RT-qPCR assay

Total RNAs were extracted from VCMs or intact insects using TRIzol reagent (Invitrogen) according to the manufacturer’s protocol. RT-qPCR assay were performed as previously described [32, 59]. The number of RGDV genome copies in the individual viruliferous *R*. *dorsalis* was calculated as the log of the copy number/μg insect RNA based on a standard curve for the RGDV P8 gene. The relative transcript expression of autophagy-related genes, *Sqstm1* gene and RGDV P8 gene in VCMs or *R*. *dorsalis* was analyzed by relative RT-qPCR assay according to the 2^−ΔΔCt^ method [59].

### Time-course detection of autophagy activation in response to viral infection

VCMs derived from *R*. *dorsalis* growing on a coverslip were inoculated with RGDV at a MOI of 1.0 for 2 h. At 12, 24, 36, 48, 72 and 96 hpi, the VCMs were collected at various time points to determine if viral infection induced autophagy pathway. Alternatively, about 500 second-instar *R*. *dorsalis* were fed on RGDV-infected rice plants for 1 day, and then transferred to healthy rice seedling. At different days padp, 100 nonviruliferous or viruliferous leafhoppers were collected at various time points to determine if viral infection can induce autophagy pathway. The activation of autophagy pathway at various time points after viral infection was analyzed by RT-qPCR and western blot assays.

### Intracellular and extracellular viral detection in VCMs

The *Ulk1*, Atg5, Atg8, *Sqstm1* and *Torc1* sequence obtained from the high-throughput transcriptome sequencing of *R*. *dorsalis* in our laboratory, and the obtained gene sequences for *Ulk1*, *Atg5*, *Atg8*, *Sqstm1* and *Torc1* of *R*. *dorsalis* were deposited in GenBank with accession numbers MF038047, MF038044, MF038045, MF038048 and MF038046, respectively. Autophagy-related *Atg5*, *Atg8* and *Torc1* genes of *R*. *dorsalis* and the *GFP* gene were amplified by RT-PCR assay. T7 RiboMAX Express RNAi System kit (Promega) was used to synthesize *in vitro* dsRNAs for these four genes according to the manufacturer’s instructions. To examine the effects of synthesized dsRNAs or the drugs (autophagy inhibitor, 3-MA; autophagy inducer, rapamycin, Woburn) on viral infection, VCMs were transfected with dsRNAs in the presence of Cellfectin (Invitrogen) or drugs for 8 h, and then inoculated with RGDV at a low MOI of 0.1. At 48 hpi, VCMs were fixed, immunolabeled with ATG8-FITC and P8-rhodamine, and then processed for immunofluorescence microscopy as described previously [57].

To further determine whether the autophagy pathway triggered by RGDV infection can facilitate viral release, VCMs were treated with dsRNAs (ds*Torc1*, ds*Atg5* or ds*GFP*) in the presence of Cellfectin for 8 h, and then inoculated with RGDV at a MOI of 10 for 2h. Then the VCMs were washed by the fresh culture medium for 3 times to remove the viruses that were not absorbed. Alternatively, to detect whether RDV infection induced autophagy pathway can facilitate viral release, VCMs were treated with 100 nM 3-MA or 20 μM rapamycin (dissolved in PBS) in the presence of Cellfectin for 8 h, and then inoculated with RDV at a MOI of 10 for 2h. Then the VCMs were washed by the fresh culture medium for 3 times to remove the viruses that were not absorbed. The supernatant of the infected cells was collected at 48 or 72 hpi and sedimented to remove cell debris [60]. Total RNAs were extracted from the supernatant using TRIzol reagent. The number of viral genome copies in the extracellular of VCMs in supernatant was calculated as described above.

### Effects of virus-induced autophagy on viral infection in leafhopper vector

Three thousands second-instar *R*. *dorsalis* were fed on RGDV-infected rice plants for 1 day, microinjected with 200 nl (0.5 μg/μl) dsRNAs (ds*Atg5*, ds*Atg8*, ds*Torc1* or ds*GFP*) using a Nanoject II Auto-Nanoliter Injector (Spring), and then kept on healthy rice seedling. At different days padp, 30 live leafhoppers were sampling daily, and continued for 18 days. Total RNA was extracted and viral genome copy was calculated as described above. In addition, at 4 days padp, the intestines of viruliferous *R*. *dorsalis* treated with dsRNAs were immunolabelled with P8-rhodamine and the ACTB dye phalloidin-FITC (Invitrogen), then examined for immunofluorescence microscopy as described previously [57].

### Transmission of RGDV by leafhoppers treated with dsRNAs

For examining the effects of autophagy pathway on viral transmission, 400 second-instar leafhoppers were microinjected with dsRNAs (ds*Atg5*, ds*Atg8*, ds*Torc1* or ds*GFP*) after they had fed on RGDV-infected rice plants for 1 day, and then kept on healthy rice seedlings. From 3 to 17 days padp, an individual insect was fed on a healthy rice seedling in one glass tube, and the rice seedlings were replaced daily. The replaced rice seedlings were grown in the greenhouse (at 25 ± 1°C, under conditions of 75 ± 5% relative humidity and a photoperiod of 16 h of light and 8 h of darkness) about 15 days to observe the appearance of disease symptoms, and then the total RNA was extracted from inoculated rice seedlings to determine the presence of transcripts for the RGDV P8 gene to calculate transmission rates. Transmission rate was calculated according to the number of positive rice plants/total number of survival rice plants fed by dsRNAs-treated leafhoppers.

### Effects of autophagy on viral infection in rice hosts

To detect whether autophagy pathway can facilitate viral infection in rice plants, 100 healthy rice seedlings (15 days old) were treated with 100 nM 3-MA for 1 day. We then inoculated the treated seedlings with RGDV for 2 days using viruliferous leafhoppers. At different days inoculation, 5 rice plants positive for transcript of RGDV P8 gene were used for assay of viral genome copies, which were calculated as the log of the copy number of P8 gene/μg rice RNA. At 4 days inoculation, relative expression levels of *Atg8* gene were detected by RT-qPCR assay as well.

### Statistical analyses

All data were analysed with SPSS, version 17.0. Percentage data were arcsine square-root transformed before analysis. Multiple comparisons of the means were conducted based on Tukey’s honest significant difference (HSD) test using a one-way analysis of variance (ANOVA). The data were back-transformed after analysis for presentation in the text and figures.

### Data availability

All relevant data are within the paper and its Supporting Information files.

## Supporting information

S1 Fig **Autophagic responses were triggered by RGDV infection in cultured leafhopper vector cells (A) and in intact insects (B).** Relative expression levels of *Ulk1*, *Atg5*, *Atg8* and *Sqstm1* genes were detected by RT-qPCR assay (panels I). The accumulation levels of ATG8, SQSTM1 and RGDV P8 were analyzed by western blot assay as well (panels II). ACTB was used as the internal control. Means (±SD) from three biological replicates are shown. The statistical significance is related to the control. *P < 0.05.
(TIF)

S2 Fig Activation of autophagy by rapamycin and ds*Torc1*. The VCMs were transfected for 8 h with PBS, rapamycin or ds*Torc1*. At 48 hpi, VCMs were immunolabeled for autophagosomes with ATG8-specific IgG conjugated to FITC (ATG8-FITC), then examined with confocal microscopy. For each condition, six different fields were observed. Bars, 10 μm.
(TIF)

S3 Fig Effects of autophagy on viral infection in rice hosts. (A) Rice plants were treated with 3-MA for 1 day, and then inoculated with RGDV for 2 days using viruliferous *R*. *dorsalis*. At different days inoculation, 5 rice plants positive for transcript of RGDV P8 gene were used for assay of viral genome copies, which were calculated as the log of the copy number of P8 gene/μg rice RNA. Means (±SD) from three biological replicates are shown. Means followed by the same lowercase letter are not significantly different (ANOVA and Tukey’s HSD test, *P* > 0.05). (B) Relative expression levels of *Atg8* gene were detected by RT-qPCR assay. Means (±SD) from three biological replicates are shown. *P < 0.05. (C) Electron microscopy showed the representative images of virus-infected rice plants. Panel II was an enlargement of the boxed area in panel I. M, mitochondrion. CW, cell wall. Bars, 500 nm.
(TIF)

S1 Table Primers used in this study. (DOC)
