# Supplementary figures and images for: Autophagy pathway induced by a plant virus facilitates viral spread and transmission by its insect vector
Source: PLoS Pathog. 2017 Nov 10;13(11):e1006727. doi: 10.1371/journal.ppat.1006727 (PMC5708841; doi:10.1371/journal.ppat.1006727)

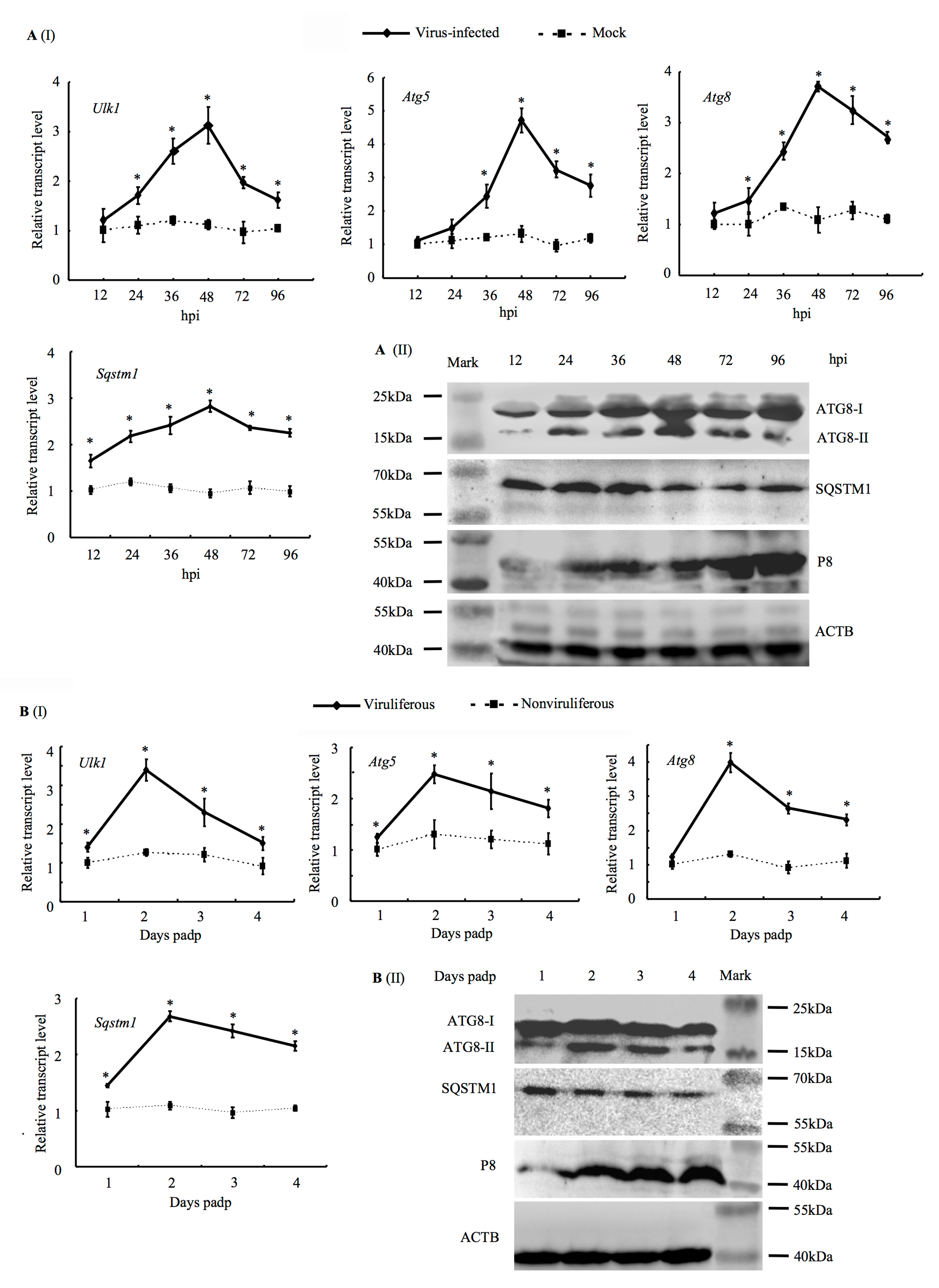

Supplement: S1 Fig — Autophagic responses were triggered by RGDV infection in cultured leafhopper vector cells (A) and in intact insects (B). Relative expression levels of Ulk1, Atg5, Atg8 and Sqstm1 genes were detected by RT-qPCR assay (panels I). The accumulation levels of ATG8, SQSTM1 and RGDV P8 were analyzed by western blot assay as well (panels II). ACTB was used as the internal control. Means (±SD) from three biological replicates are shown. The statistical significance is related to the control. *P < 0.05. (TIF) [file ppat.1006727.s001.tif]

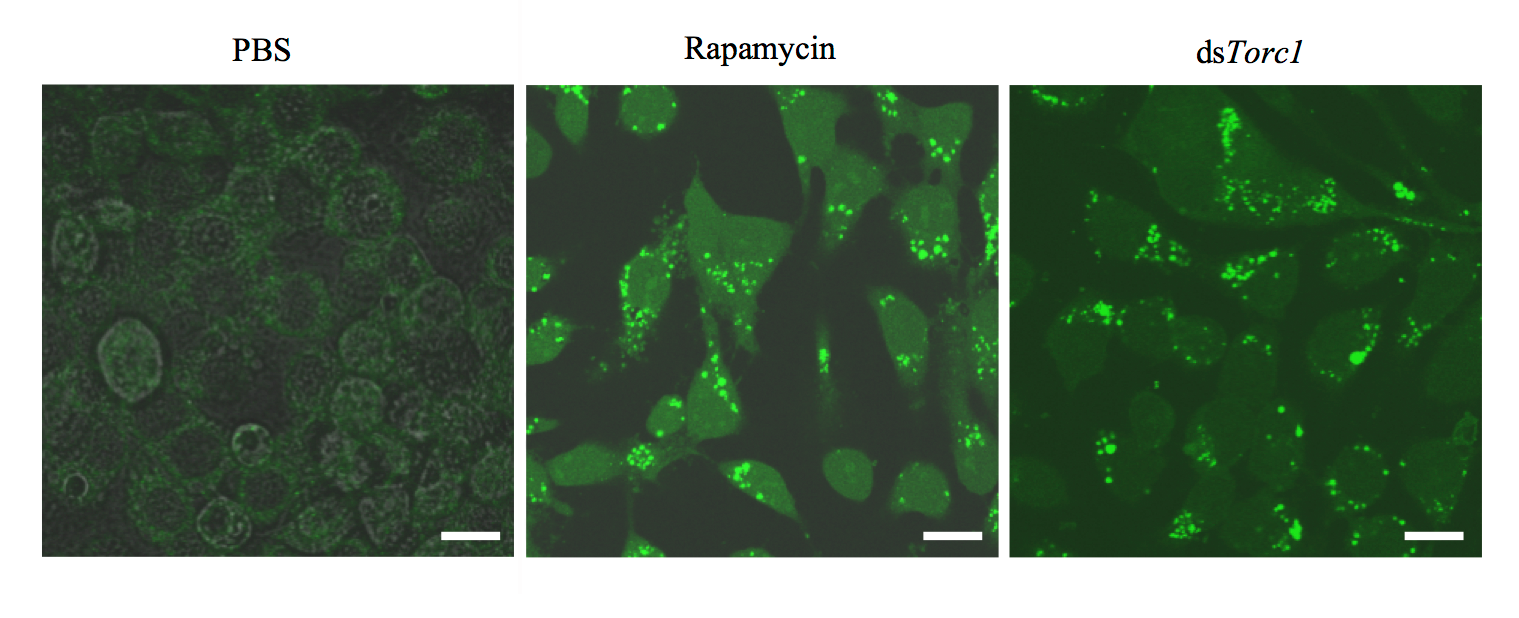

Supplement: S2 Fig — The VCMs were transfected for 8 h with PBS, rapamycin or dsTorc1. At 48 hpi, VCMs were immunolabeled for autophagosomes with ATG8-specific IgG conjugated to FITC (ATG8-FITC), then examined with confocal microscopy. For each condition, six different fields were observed. Bars, 10 μm. (TIF) [file ppat.1006727.s002.tif]

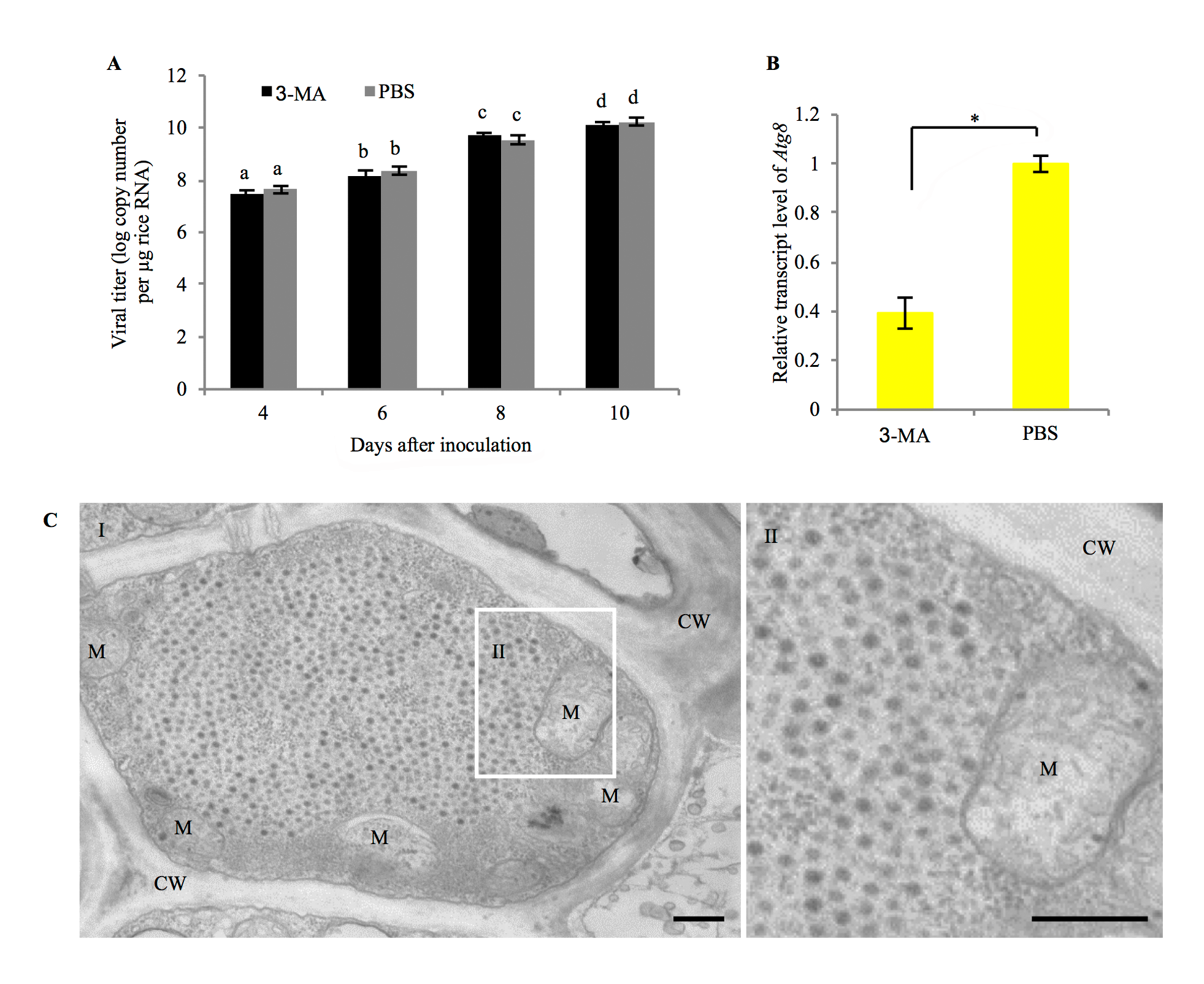

Supplement: S3 Fig — (A) Rice plants were treated with 3-MA for 1 day, and then inoculated with RGDV for 2 days using viruliferous R. dorsalis. At different days inoculation, 5 rice plants positive for transcript of RGDV P8 gene were used for assay of viral genome copies, which were calculated as the log of the copy number of P8 gene/μg rice RNA. Means (±SD) from three biological replicates are shown. Means followed by the same lowercase letter are not significantly different (ANOVA and Tukey’s HSD test, P > 0.05). (B) Relative expression levels of Atg8 gene were detected by RT-qPCR assay. Means (±SD) from three biological replicates are shown. *P < 0.05. (C) Electron microscopy showed the representative images of virus-infected rice plants. Panel II was an enlargement of the boxed area in panel I. M, mitochondrion. CW, cell wall. Bars, 500 nm. (TIF) [file ppat.1006727.s003.tif]
