# Supplementary material for: Autophagy pathway induced by a plant virus facilitates viral spread and transmission by its insect vector
Source: PLoS Pathog. 2017 Nov 10;13(11):e1006727. doi: 10.1371/journal.ppat.1006727 (PMC5708841; doi:10.1371/journal.ppat.1006727)
Supplement: S1 Table — (DOC) [file ppat.1006727.s004.doc]

**Table 1S.** Primers used in this study.

| Oligonucleotides | Sequence (5′-3′) |
| --- | --- |
| Primers used for dsRNA synthesis | |
| T7-*R. dorsalis*-*Atg5*-F | T7-TCCAACCTCCAGAACCAT |
| T7-*R. dorsalis*-*Atg5*-R | T7-GGCTTCATCGGGAAACAC |
| T7-*R. dorsalis*-*Atg8*-F | T7-GAAGGCAAGAATCGGTGATCT |
| T7-*R. dorsalis*-*Atg8*-R | T7-TTCGTGGTGCTCCTGGTAGA |
| T7-*R. dorsalis*-*Torc1*-F | T7-GTTCATCCAACCCAAACCCTG |
| T7-*R. dorsalis*-*Torc1*-R | T7-TACCGTGCATGTGCCTCTATC |
| T7-*GFP*-F | T7-CTTGTTGAATTAGATGGTGATGTT |
| T7-*GFP*-R | T7-TTTCGAAAGGGCAGATTGT |
| T7, ATTCTCTAGAAGCTTAATACGACTCACTATAGGG | |
| Primers used for RT-qPCR | |
| q*R. dorsalis*-*ACTB*-F | AGAAGTCCTACGAGTTGCCTGATG |
| q*R. dorsalis*-*ACTB*-R | TTCATGATGGAGTTGTAGACGGTC |
| q*N. cincticeps*-*ACTB*-F | GGGATACAGTTTCACCACG |
| q*N. cincticeps*-*ACTB*- R | GACACCTGAATCGCTCGT |
| q*R. dorsalis*-*Ulk1*-F | CCCCTCAGCCTCAACATA |
| q*R. dorsalis*-*Ulk1*-R | GGTCCCTAAACTCCACAGAT |
| q*R. dorsalis*-*Atg5*-F | ACTCCAACCTCCAGAACCAT |
| q*R. dorsalis*-*Atg5*-R | CCATTTCAAAGGCAGTCCAT |
| q*R. dorsalis*-*Atg8*-F | AGAAGTATCTGGTGCCCTCCG |
| q*R. dorsalis*-*Atg8*-R | CTTCGTGGTGCTCCTGGTAGA |
| q*R. dorsalis*-*Sqstm1*-F | CCGTAAACCATATTCTATACCC |
| q*R. dorsalis*-*Sqstm1*-R | CTCCGAGACCAACTCAAGC |
| qRGDV-P8-F | AACGAGTACAAATTGAGACCCTAAC |
| qRGDV-P8-R | TGAGCAGGAACTTCACGACAAC |
| qRDV-P8-F | GTTCGGTGCAACGGAGATAC |
| qRDV-P8-R | TCTGAAACGGTAGGCTTGGT |
| Primers used to detect RGDV or RDV P8 gene | |
| RGDV-P8-F | ATGTCGCGCCAAGCTTG |
| RGDV-P8-R | CTCAGTCAAAGTGTTCATCGACT |
| RDV-P8-F | CCATCCGATACATGTCACGCCAGATGTGGTTAG |
| RDV-P8-R | CTCAGTCAAAGTGTTCATCGACT |
